# The Floating Knee in Pediatric Patients: A Single-Center Retrospective Study From a Referral Center

**DOI:** 10.7759/cureus.29517

**Published:** 2022-09-23

**Authors:** Alberto Daniel Navarro Vergara, Alberto N Fretes

**Affiliations:** 1 Orthopedics and Traumatology, Hospital de Trauma “Manuel Giagni”, Asunción, PRY; 2 Pediatric Orthopedics, Hospital de Trauma “Manuel Giagni”, Asunción, PRY

**Keywords:** outcomes, floating knee, fracture, tibia, femur

## Abstract

Introduction

An ipsilateral fracture of the femur and tibia (also known as floating knee) is a rare injury in pediatric patients. Recent advances rapidly made the use of intramedullary elastic nails the standard of care in the management of long bone fractures, including floating knee injuries, in patients with immature skeletons. Currently, we have observed a trend of fixing both fractured bones, thus improving functional outcomes, and reducing sequelae. The aim of this study was to report our experience in the management and functional outcomes of floating knee injuries in a single trauma-I level center.

Materials and methods

This is a retrospective study of consecutive cases from June 1, 2018, to March 31, 2022, from a single trauma center. Preoperative and postoperative records and radiographs were assessed, including the epidemiological data and characteristics of the fractures type of treatment, mechanism of injury, complications, and functional evaluation at the last follow-up, which was performed according to the criteria described by Karlstrom and Olerud.

Results

Twenty-five patients were included during the four years of study, of which 18 were male (72%), and the mean age was 9.6 years. Overall, 96% of the cases were related to traffic accidents. An analysis of the provided treatment showed that 19 cases (76%) were resolved with surgery on at least of the bones, and six cases were managed with simultaneous orthopedic treatments in both injuries. Excellent outcomes were achieved in 15 cases (60%), good outcomes in three, fair outcomes in five, and poor outcomes in one. Concerning the classification of injuries according to the criteria developed by Letts and Vincent,it was observed that type D was the most frequent one. With regard to exposed bone injuries, 15 cases presented with open fractures. Complications were found in eight (32%) cases, of which five were related to limb discrepancy and three with functional limitations of the knee, with changes in its range of motion. There was also one case of nonunion of the distal tibia.

Conclusion

Traffic accidents are the main cause of floating knees. Surgical management of the fractures brought satisfactory outcomes and reduced complications. Thus, fixation of injuries is recommended for early return to daily activities and for a reduction in residual complications.

## Introduction

Isolated femoral and tibial fractures are frequent causes of hospitalization in the pediatric unit of referral trauma centers. However, simultaneous ipsilateral femoral and tibial fractures, described as "floating knees" are rare events in pediatric patients [1-3].

Recent advances rapidly promoted the use of intramedullary elastic nails for the management of long bone fractures in patients with immature skeletons, gradually replacing the historical treatment of these injuries using traction and cast with prolonged hospitalization and recovery [2,4,5].

In addition to its infrequency, "pediatric floating knee" presents different patterns and characteristics when compared to the same injury in the adult population, especially in terms of associated injuries, management, and prognosis [6-8]. Several studies have been advocating conservative management of this injury in immature skeletons; however, clinical and radiographic outcomes are generally poor [9,10]. Currently, there is a trend to fix both fractures or at least the femur fracture, thus improving functional outcomes and reducing sequelae [1,2,5,6].

The primary aim of this study was to report our experience with pediatric floating knee injuries, describing treatment methods and presenting clinical outcomes. The secondary aims were to describe the characteristics and the pattern of fractures as well as the causative trauma mechanisms and to report its complications.

## Materials and methods

After approval was obtained from the Teaching Committee and the Ethics Committee of the Hospital de Trauma “Manuel Giagni” (Number 1336), a retrospective study was conducted to assess all consecutive cases of ipsilateral femur and tibia fractures in the immature skeletons. All patients admitted from June 1, 2018, to May 31, 2022 aged 16 years old or less were included. The patients' medical records were evaluated, and identities were protected. Cases with missing documentation or fractures of bones with an underlying disease, either neoplasm or previous infections, were excluded.

Medical records and radiographs were analyzed, and the following data were obtained: patients’ demographics, mechanism of injury and affected side, type of treatment (non-operative or operative), and type of implant (intramedullary (either flexible or rigid) or plate) (Table 1).

**Table 1:** Age, sex, mechanism of trauma, and treatment received. ESIN, elastic stable intramedullary nailing; ORIF, open reduction and internal fixation

Age	Sex	Mechanism	Treatment Femur	Treatment Tibia
0	Male	Fall from motorcycle	Pavlik harness	Conservative cast
0	Male	Direct trauma	Spica cast	Cast
0	Female	Fall from motorcycle	Pavlik harness	Pavlik harness
0	Male	Fall from motorcycle	Pavlik harness	Pavlik harness
2	Male	Automotive vehicle	Spica cast	Spica cast
2	Male	Fall from motorcycle	Spica cast	Cast
6	Female	Fall from motorcycle	ESIN	ESIN
7	Female	Fall from motorcycle	ESIN	External fixator
8	Female	Fall from motorcycle	ESIN	Cast
8	Female	Fall from motorcycle	Bridge plating	ESIN
11	Male	Fall from motorcycle	ESIN	ORIF with plate and screws
11	Female	Fall from motorcycle	External fixator	External fixator
11	Male	Fall from motorcycle	ORIF with plate and screws	ESIN
13	Female	Fall from motorcycle	Rigid retrograde intramedullary nail	Rigid intramedullary nail
14	Male	Fall from motorcycle	ORIF with screws	ORIF with screws
14	Male	Fall from motorcycle	Rigid antegrade intramedullary nail	ORIF with screws
14	Male	Fall from motorcycle	Rigid antegrade intramedullary nail	Rigid intramedullary nail
14	Male	Fall from motorcycle	Rigid retrograde intramedullary nail	Rigid intramedullary nail
14	Male	Fall from motorcycle	Rigid retrograde intramedullary nail	Rigid intramedullary nail
14	Male	Fall from motorcycle	Rigid retrograde intramedullary nail	Rigid intramedullary nail
15	Male	Fall from motorcycle	Rigid antegrade intramedullary nail	Rigid intramedullary nail
15	Male	Fall from motorcycle	Rigid antegrade intramedullary nail	ESIN
15	Male	Fall from motorcycle	Rigid antegrade intramedullary nail	Rigid intramedullary nail
16	Male	Fall from motorcycle	Rigid retrograde intramedullary nail	Rigid intramedullary nail
16	Male	Fall from motorcycle	ORIF with screws	External fixator

In the case of non-operative treatment, the use of a cast or traction was also recorded. For radiographic evaluation, fracture union was defined as the presence of bone bridge in at least three of four cortices in both radiographic views, considering each affected bone independently [10], which allowed patients to resume weight bearing without support during outpatient follow-ups, as reported in follow-up records. Patients were clinically and radiographically followed at two, four, and eight weeks and then three, six, and 12 months post-operatively, and then once a year. The function was assessed according to the criteria described by Karlstrom and Olerud (Table 2) [11].

**Table 2:** Evaluation of outcomes. The function was assessed as per the criteria described by Karlstrom and Olerud [11].

Criterion	Excellent	Good	Fair	Poor
Subjective symptoms thigh/leg	None	Mild and intermittent	Symptoms affect functionality	Major functional limitation, pain at rest
Subjective symptoms knee/ankle	None	Mild and intermittent	Symptoms affect functionality	Major functional limitation, pain at rest
Ability to walk	Intact	Mild and intermittent	Limitations for long distances	Requires help
Sports	Normal	Not all sports	Minimum sports	No sports
Angulations/rotation	0	< 10 degrees	10 to 20 degrees	> 20 degrees
Shortening	0	< 1 centimeter	1 to 3 cm	> 3 centimeters
Joint stiffness/limitations	0	< 10 degrees in hip, knee, ankle	10 to 20 degrees in ankle, 20 to 40 degrees in hip, knee, or both	> 20 degrees in ankle

**Figure 1:**
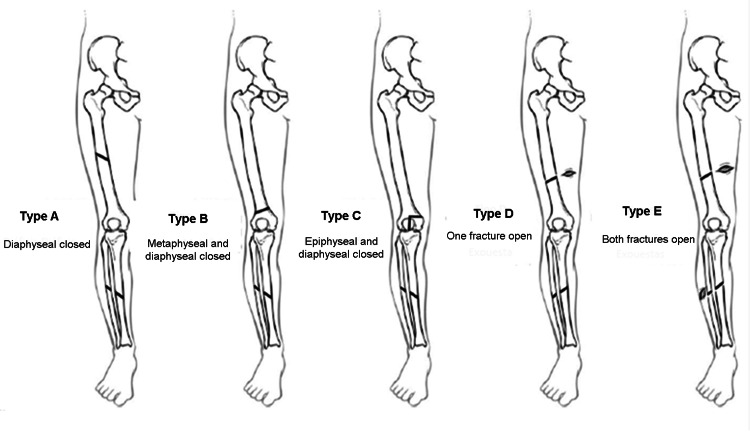
Letts et al. classification Taken from Letts et al. [4]. Reproduced with permission of the original publisher.

Floating knee injuries were classified according to the system proposed by Letts et al. [4] (Figure 1). Isolated fracture patterns (femur and tibia) were classified according to the AO/OTA system [12-14]. Open fractures were classified according to the system described by Gustillo and Anderson [13].

The total length of hospital stay and the presence of associated injuries were recorded. Associated injuries were divided into four categories (skull, chest, abdomen/pelvis, and extremities). Secondary procedures, such as surgical debridement, soft-tissue coverage, or implant removal, were also recorded.

Descriptive statistics were used to summarize the characteristics of our patient data.

## Results

During the period of the study, 25 patients with floating knee injuries were treated at our institution. Of these, 18 (72%) were male with a mean age of 9.6 years (ranging from 0 to 16 years old). The injury most often occurred at the age of 14 years (24%). Remarkably, four (16%) cases involved children younger than 12 months of age. Additionally, 24 (96%) cases were related to traffic accidents, of which 22 (88%) involved falls from motorcycles; and 16 (64%) cases occurred on the left side. Table 1 presents the demographic data of the 25 patients.

Of the 25 patients, 19 (76%) patients had at least one bone surgically fixed, and six (24%) patients were managed non-operatively. Of the 19 patients operated on, 18 had fixations on both femur and tibia fracture, and one was managed with internal fixation of the femur fracture and non-operative treatment for the tibia fracture (Case 5). Of the 19 patients treated with surgical fixation of the femur fracture, intramedullary implants were used in 14 cases (10 patients with rigid intramedullary nails and four patients with elastic stable intramedullary nails - ESIN). All ESIN fixations were performed through a retrograde technique [15-22]. Two cases were managed with a plate, two cases with lag-screws only, and one case with external fixation. Of the 18 treated with surgical fixation of the tibia fracture, a rigid intramedullary nail was used in eight, ESIN in four, external fixation in three, lag-screws only in two, and a plate in one case. Of the six patients managed non-operatively, three were treated with a Pavlik harness, one with a boot cast for the management of the tibial fracture, two with a spica cast, and one with a long leg cast to stabilize both injuries. Table 1 specifies the treatment performed for each patient.

Outcome evaluation was performed on all patients in the last outpatient consultation. Using the criteria described by Karlstrom and Olerud, 15 (60%) patients were scored excellent, three (12%) good, six (24%) fair, and one (4%) poor. The poor outcome was observed in a patient with severe trauma, presenting extensive soft tissue injuries and delayed severe bone infection of the distal tibia, requiring multiple debridements and soft tissue coverage with a local flap. The patient is currently being followed up by the team.

**Figure 2:**
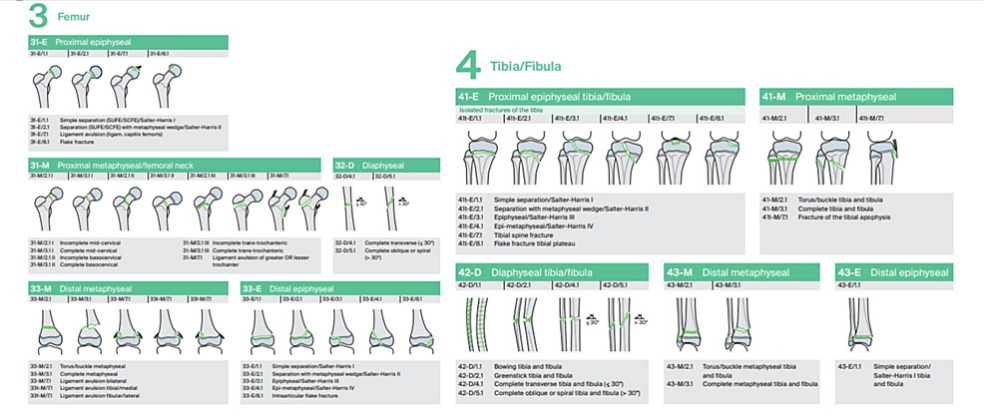
AO pediatric comprehensive classification of long bone fractures Source: Slongo et al. [14] Reproduced with permission of the original publisher.

According to the criteria proposed by Letts et al. [4], 10 (40%) cases were classified as type D, five (20%) cases as type E, five (20%) as type A, 4 (16%) as type B, and 1 (4%) as type C. Fifteen (60%) patients had at least one open fracture. All open femur fractures (n = 7) were classified as Gustilo and Anderson grade IIIA. Six open tibia fractures were classified as Gustilo and Anderson grade II, one as grade IIIA, and one as grade IIIB. Eight open fractures classified as Letts et al. type D involved the tibia and five involved both the femur and tibia. No patient had vascular injuries requiring repair. There was a predominance of shaft femur and tibia fractures. A femur shaft fracture was observed in 20 (80%) cases, and a tibia shaft fracture was seen in 18 (72%) cases. Simultaneous diaphyseal occurred in 14 (56%) cases. Fracture patterns were classified according to the AO/OTA guidelines for pediatric injuries (Figure 2).

Associated injuries were found in 14 (54%) patients. Of these, there were nine skull injuries, with no severe traumatic brain injury, one pulmonary contusion requiring thoracic drainage, one pelvic fracture managed non-operatively, and one proximal ulna fracture treated with a long arm cast. One patient progressed to compartment syndrome of the thigh, which was resolved with immediate fasciotomy and external fixation of the fracture up to fracture union. Length of hospital stay ranged from two to 40 days, with a mean of 16 days. Eighteen (72%) cases required secondary procedures such as debridement and plastic surgery for coverage.

**Figure 3:**
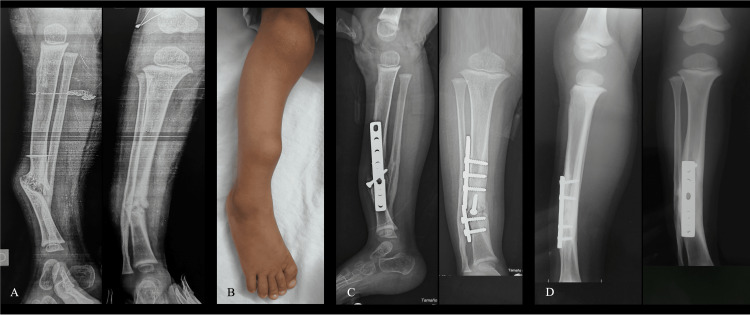
Nonunion of the distal tibia (Case 1) A and B: Radiographs and clinical aspects of a patient managed with a cast. Observe the hypertrophic nonunion with varus and antecurvatum deformity. C: The patient was submitted to open reduction and internal fixation with a plate, however, an implant failure occurred and the fracture did not heal. D and E: The patient was revised with new osteosynthesis with a plate. An autogenous bone graft was used. X-rays in the last follow-up show uneventful healing of the fracture with slight, acceptable residual varus.

There were eight (32%) complications; five patients presented a residual lower limb discrepancy and three had reduced motion of the ipsilateral knee. One patient with a nonunion of the distal tibia was resolved with a new surgery open reduction, bone graft, and fixation with a plate (Figure 3).

## Discussion

Currently, there is no consensus on the optimal management of floating knee injuries in pediatric patients, mostly because of its rarity [1-3,19,22]. Almost 50 years have passed since the first case was reported by Blake et al. [4,23], who described high energy as the main cause of this type of injury. Currently, there is a trend for stabilizing long bone fractures even in the immature population, as this has been shown advantageous in terms of both rehabilitation and outcome. Indeed, Yue et al. [5] observed a reduction in residual complications, such as angle deviations or limb-length discrepancy, when these fractures were treated surgically. Relative stability is highly preferred as the standard treatment with excellent outcomes and early return to daily activities [15-22]. Even though further studies are needed to confirm that this treatment is beneficial for patients with ipsilateral injuries of the femur and tibia, the Letts et al. [4] study group recommended that at least one of the injuries should be fixed to promote stability to the affected extremity. Yue et al. [5] also suggested that surgical treatment must be done at least for the femur in cases of floating knees. In developed countries, few cases of floating knees are managed with prolonged tractions or with cast immobilization [1], since technology and surgical supplies developed for use in the immature skeleton are easily available. In our study, 19 patients were operated on, and non-operative treatment was used for younger patients, mostly for the tibia. Of relevance, 16% of our cases involved children younger than 12 months of age. Intramedullary fixation was the most used treatment option among surgeons in our hospital. One of the potential reasons for this was the location of the fracture line, most of them occurring in the shaft of both the femur and tibia.

Our results were similar to those published by the CORTICES study [1] in 2019, which found that the high-energy mechanisms involved in traffic accidents are widely responsible for the floating knee. In the CORTICES study, 24 of the 25 cases were related to this type of accident, of which 22 resulted from falls from motorcycles after impact with an automobile vehicle, other motorcycles, or a fixed object. The mechanism of injury is of great relevance, as many patients are polytraumatized or have more than one skeletal injury. In our series, associated injuries were found in 14 patients, nine of them had mild to moderate traumatic brain injuries, reflecting the lack of basic protective equipment in the use of wheeled vehicles, such as car seatbelts or motorcycle helmets.

**Figure 4:**
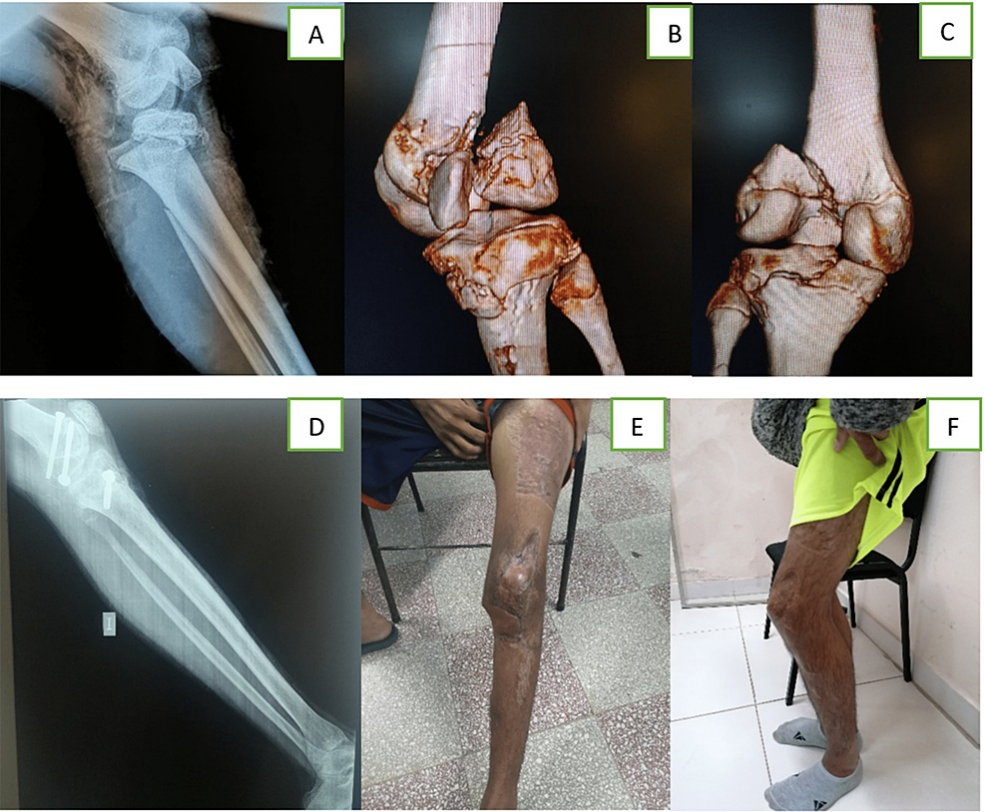
Clinical Case (Case 14) A, B, C: Salter-Harris Type IV femoral injury and Salter-Harris III tibial injury. D: immediate postoperative where both fractures managed by open reduction and internal fixation with lag screws. E and F: Three-month and 30-month follow-up showing satisfactory, but not perfect recovery. There is residual knee flexion. In the CT scans above, note the patella within the distal femur fracture.

We found satisfactory outcomes in 18 patients (15 excellent and three good), which can be partially attributed to the fixation strategy. Seven patients had unsatisfactory outcomes, with bone healing disturbances and inadequate regain of range of motion of the ipsilateral knee being the major reasons for that. Leg-length discrepancies were seen in five patients and nonunion in one. We believe that good quality reduction of these fractures, with the anatomical alignment of the axis, and stable fixation are the keys to reducing the number and the severity of these complications. In addition, the early introduction of a rehabilitation protocol is facilitated when both fractures are stabilized adequately, which better controls pain and reduced the risk of loss of reduction. Even injuries with joint involvement, which are infrequent in the floating knee association (Figure 4), can be better managed this way, thus reducing the risk of late sequelae. In our cases, in patients with expected little growth potential, the management was performed with implants commonly used in adults (femoral and tibial rigid nails).

The limitations of this study include its retrospective nature and the relatively short follow-up, which lasted for only six months in some cases. Since a longer time is required to identify some late complications, such as angle deviations or premature physeal closure, all patients are followed up in our institution.

One of the strengths of the study is the fact that all the cases described were managed in the same hospital. Despite the decision to operate or not, or that using IM or extramedullary devices in patients was a prerogative of the surgeon who was in the hospital and/or operating room on the day, all cases are discussed every day, so there is a trend to follow certain recommendations existing in the most updated literature. Another strength of our study was the adequate follow-up, with no loss of patients during the period of the study.

## Conclusions

In conclusion, this study shows again that traffic accidents are almost the only responsible cause for floating knee injuries and that surgical management of fractures brings satisfactory outcomes and reduces the percentage of severe complications. Therefore, similar to other authors, we recommend the fixation of injuries for an early return to daily activities and reduction of residual complications.

## Appendices

We have the corresponding permission for the use of the figures.

## References

[1] (2019). The pediatric "floating knee" injury: a state-of-the-art multicenter study. J Bone Joint Surg Am.

[2] Anari JB, Neuwirth AL, Horn BD, Baldwin KD (2017). Ipsilateral femur and tibia fractures in pediatric patients: A systematic review. World J Orthop.

[3] Nakaniida A, Sakuraba K, Hurwitz EL (2014). Pediatric orthopaedic injuries requiring hospitalization: epidemiology and economics. J Orthop Trauma.

[4] Letts M, Vincent N, Gouw G (1986). The "floating knee" in children. J Bone Joint Surg Br.

[5] Yue JJ, Churchill RS, Cooperman DR, Yasko AW, Wilber JH, Thompson GH (2000). The floating knee in the pediatric patient. Nonoperative versus operative stabilization. Clin Orthop Relat Res.

[6] Arslan H, Kapukaya A, Kesemenli C, Subaşi M, Kayikçi C (2003). Floating knee in children. J Pediatr Orthop.

[7] Rollo G, Falzarano G, Ronga M (2019). Challenges in the management of floating knee injuries: Results of treatment and outcomes of 224 consecutive cases in 10 years. Injury.

[8] Feron JM, Bonnevialle P, Pietu G, Jacquot1 F (2015). Traumatic floating knee: a review of a multi-centric series of 172 cases in adult. Open Orthop J.

[9] Hedequist D, Starr AJ, Wilson P, Walker J (1999). Early versus delayed stabilization of pediatric femur fractures: analysis of 387 patients. J Orthop Trauma.

[10] Whelan DB, Bhandari M, Stephen D, Kreder H, McKee MD, Zdero R, Schemitsch EH (2010). Development of the radiographic union score for tibial fractures for the assessment of tibial fracture healing after intramedullary fixation. J Trauma.

[11] Karlström G, Olerud S (1977). Ipsilateral fracture of the femur and tibia. J Bone Joint Surg Am.

[12] Meinberg EG, Agel J, Roberts CS, Karam MD, Kellam JF (2018). Fracture and dislocation classification compendium-2018. J Orthop Trauma.

[13] Gustilo RB, Anderson JT (1976). Prevention of infection in the treatment of one thousand and twenty-five open fractures of long bones: retrospective and prospective analyses. J Bone Joint Surg Am.

[14] Slongo T, Audigé L, Schlickewei W, Clavert JM, Hunter J (2006). Development and validation of the AO pediatric comprehensive classification of long bone fractures by the Pediatric Expert Group of the AO Foundation in collaboration with AO Clinical Investigation and Documentation and the International Association for Pediatric Traumatology. J Pediatr Orthop.

[15] Ligier JN, Metaizeau JP, Prévot J, Lascombes P (1988). Elastic stable intramedullary nailing of femoral shaft fractures in children. J Bone Joint Surg Br.

[16] Flynn JM, Bashyal RK, Yeger-McKeever M, Garner MR, Launay F, Sponseller PD (2011). Acute traumatic compartment syndrome of the leg in children: diagnosis and outcome. J Bone Joint Surg Am.

[17] Fraser RD, Hunter GA, Waddell JP (1978). Ipsilateral fracture of the femur and tibia. J Bone Joint Surg Br.

[18] Veith RG, Winquist RA, Hansen ST Jr (1984). Ipsilateral fractures of the femur and tibia. A report of fifty-seven consecutive cases. J Bone Joint Surg Am.

[19] Rethnam U, Yesupalan RS, Nair R (2007). The floating knee: epidemiology, prognostic indicators &amp; outcome following surgical management. J Trauma Manag Outcomes.

[20] Anastopoulos G, Assimakopoulos A, Exarchou E, Pantazopoulos T (1992). Ipsilateral fractures of the femur and tibia. Injury.

[21] Al-Mahdi W, Ibrahim MM, Spiegel DA, Arkader A, Nance M, Baldwin K (2020). Is systemic inflammatory response syndrome relevant to pulmonary complications and mortality in multiply injured children?. J Pediatr Orthop.

[22] Baldwin K, Hsu JE, Wenger DR, Hosalkar HS (2011). Treatment of femur fractures in school-aged children using elastic stable intramedullary nailing: a systematic review. J Pediatr Orthop B.

[23] Blake R, McBryde A Jr (1975). The floating knee: Ipsilateral fractures of the tibia and femur. South Med J.

